# Surveillance for severe hand, foot, and mouth disease from 2009 to 2015 in Jiangsu province: epidemiology, etiology, and disease burden

**DOI:** 10.1186/s12879-018-3659-7

**Published:** 2019-01-22

**Authors:** Hong Ji, Huan Fan, Peng-xiao Lu, Xue-Feng Zhang, Jing Ai, Chao Shi, Xiang Huo, Chang-jun Bao, Jun Shan, Yu Jin

**Affiliations:** 1 Medical School of Nanjing University, Nanjing, 210093 China; 2 Department of Acute Infectious Disease Control and Prevention, Jiangsu Province Center for Disease Control and Prevention, Nanjing, 210009 China; 3 The Affiliated Children’s Hospital of Nanjing Medical University, Nanjing, 210008 China; 4 Wuxi Municipal Center for Disease Control and Prevention, Wuxi, 214023 China

**Keywords:** Severe hand, foot and mouth disease, Epidemiology, Disease burden, CNS complication, Enterovirus A71, Coxsackievirus A16, Coxsackievirus A6, Coxsackievirus A10, Pathogen spectrum

## Abstract

**Background:**

Severe hand, foot, and mouth disease (HFMD) is a common childhood illness caused by various enteroviruses. The disease has imposed increased burden on children younger than 5 years old. We aimed to determine the epidemiology, CNS complication, and etiology among severe HFMD patients, in Jiangsu, China.

**Methods:**

Epidemiological, clinical, and laboratory data of severe HFMD cases were extracted from 2009 to 2015. The CNS complication, annually severe illness rates, mortality rates, severity-PICU admission rates, severity-hospitalization rates, and so on were analyzed to assess the disease burden of severe HFMD. All analyses were stratified by time, region, population, CNS involvement and serotypes. The VP1 gene from EV-A71, CV-A16, CV-A6, CV-A10 and other enteroviruses isolates was amplified. Phylogenetic analysis was performed using MEGA5.0.

**Results:**

Seven thousand nine hundred ninety-four severe HFMD cases were reported, of them, 7224 cases were inpatients, 611 were PICU inpatients, and 68 were fatal. The average severe illness rate, mortality rate, severity−fatality rate, severity-PICU admission rate, and severity-hospitalization rate were 14.54, 0.12,8506, 76,430, and 903,700 per 1 million, respectively. The severe illness rate was the highest in the 12–23 months age group, and the greatest mortality rate was in the 6–11 months age group. Geographical difference in severe illness rate and mortality were found. Patients infected with EV-A71 were at a higher proportion in different CNS involvement even death. EV-A71, CV-A16 and other enteroviruses accounted for 79.14, 6.49, and 14.47%, respectively. A total of 14 non-EV-A71/ CV-A16 genotypes including CV-A2, CV-A4, CV-A 6, CV-A9, CV-A10, CV-B1, CV-B2, CV-B3, CV-B4, CV-B5, E-6, E-7, E-18, and EV-C96 were identified. Phylogentic analyses demonstrated that EV-A71 strains belonged to subgenotype C4a, while CV-A16 strains belonged to sub-genotype B1a and sub-genotype B1b of genotype B1. CV-A6 strains were assigned to genogroup F, and CV-A10 strains belonged to genogroup D.

**Conclusions:**

Future mitigation policies should take into account the age, region heterogeneities, CNS conditions and serotype of disease. Additional a more rigorous study between the mild and severe HFMD should be warranted to elucidate the difference epidemiology, pathogen spectrum and immunity patterns and to optimize interventions in the following study.

**Electronic supplementary material:**

The online version of this article (10.1186/s12879-018-3659-7) contains supplementary material, which is available to authorized users.

## Background

Hand, foot, and mouth disease (HFMD) is a common childhood disorder that typically presents as a brief, febrile illness characterized by the association of fever, skin eruptions on hands and feet, and vesicles in the mouth. The majority of HFMD cases are mild or typically asymptomatic, but severe and potentially life-threatening central nervous system (CNS) complication such as encephalitis, meningitis, acute flaccid paralysis (AFP), myocarditis, and pulmonary edema have also been reported [1–3]. Severe HFMD outbreaks with fatal cardio-pulmonary and neurologic complications have increasingly occurred in the Asian-Pacific region since 1997 and have attracted attention from many countries [4–9].

Various enteroviruses (EVs) could cause HFMD, including human Enterovirus A 71 (EV-A71), EV-A76, EV-A89, EV-A90, EV-A91, EV-A92, EV-A 114, Coxsackievirus A16 (CV-A16), CV-A6, CV-A10, CV-A12, CV-A14, CV-A21, CV-A22, Coxsackievirus B1 (CV-B1), CV-B2, CV-B3, CV-B4, CV-B5,Echovirus 3 (E-3), E-6, E-7, E-9, E-11, E-13, E-16, and E-30 [10–15]. Among them, EV-A71 is the dominant pathogen causing severe HFMD manifesting neurological complication [16–18]. Besides, HFMD outbreaks with severe cases caused by other EVs such as CV-A6 and CV-A10 have been increasingly identified [19], highlighting the importance of non-EV-A71/CV-A16 EVs and the necessity of surveillance of aetiological agents of severe HFMD.

In the past decade, countries in the Asia-Pacific region have experienced the increasing occurrence of HFMD outbreaks. However, these reports were mostly descriptive [20–22], the indicator of incidence was mostly used to assess all HFMD disease burden,with very few exceptions that assessed severity. In the present study, we used the CNS complication composition, multiple epidemiological evaluation indicators such as severe illness rates, mortality, severity-PICU(Paediatric Intensive Care Unit) admission rates,,severity-hospitalization rates, to conduct a comprehensive assessment the severity of HFMD disease. In addition, we used phylogenetic analysis to determine the molecular epidemiology of EV-A71, CV-A16,CV-A6, and CV-A10. Virus isolation and DNA sequencing of PCR products of VP1 and the 5′ non-translated region were also performed to identify the serotype of the non-EV-A71/CV-A16/CV-A6/CV-A10 EVs. Our results will not only serve as a prevaccination baseline against which future interventions comparedand but also provide a better understanding of the viral etiology of severe HFMD and the scientific basic for developing EV-A71/CV-A16/CV-A6 vaccines or multivalent vaccines in the further.

## Methods

### Case definitions

In this study, the clinical diagnosis of HFMD followed the criteria issued by National Health and Family Planning Commission of the People’s Republic of China. A probable severe case of HFMD was defined as if presenting of symptoms/signs of HFMD at least one of the following neurological complications: aseptic meningitis, encephalitis, encephalomyelitis, acute flaccid paralysis(AFP), autonomic nervous system dysregulation, or cardiopulmonary complications (pulmonary oedema, pulmonary haemorrhage, or cardiorespiratory failure). The diagnosis of above neurological complications were based on the guide to clinical management and public health response for HFMD by World Health Organization [23] (http://apps.who.int/iris/handle/10665/207490). The clinical criteria for diagnosis and notification of severe HFMD were made available to all medical practitioners [24]. A confirmed case was defined as a probable case with laboratory evidence of enterovirus infection detected by real-time reverse transcription PCR (real-time RT-PCR) (ie: EV-A71, CV-A16, or other non-EV-A71/CV-A16)in stool samples, throat and rectal swabs .

### Epidemiological, clinical information and specimen collection

A total of 7994 probable and confirmed severe cases were reported online to the China center for disease control and prevention (CDC) within 24 h after diagnosis with, a standardized questionnaire of severe HFMD including demographic, epidemiological, and clinical information collected. According to the HFMD Monitoring Technology Plan in China, throat swabs, rectal swabs, or stool specimens were collected from every severe and fatal case. All samples were transported to the laboratory using a cold chain (under 0 °C) within 24 h and stored in − 80 °C for testing within 1 month.

### RNA extraction and real-time RT-PCR

Total RNA was extracted from 50 μl of above samples using 5 × MagMAXTM − 96 Viral Isolation Kit (Ambion, USA) according to the manufacturer’s instructions. The real-time RT-PCR was performed to detect the presence of the universal sequence of enterovirus and the specific sequences of EV-A71, CV-A16, CV-A6 or CV-A10 were detected with commercial kit (JC20302 and JC20205, BioPerfectus technologiens, Jiangsu, China) according to the manufacturer’s protocols.

### Virus isolation and sequencing

EV-A71, CV-A16, CV-A6, CV-A10 and non-EV-A71/CV-A16/CV-A6/CV-A10 RNA positive samples were inoculated into human rhabdomyosarcoma (RD) cell lines in Minimum essential medium(MEM) supplemented with 10% fetal bovine serum (FBS), 100 IU of penicillin, and 100 μg of streptomycin, and cultured at 37 °C for 5 to7 days. The culture medium and cells were collected when cytopathic effect (CPE) was observed. The entire VP1 genes of the EV-A71 and CV-A16 stains isolated were amplified by RT-PCR using in-house primers that flanked the VP1 gene: EV-A71-VP1-F: 5`- TATAATAGCACTAGCGGCAGC-3`(nucleotides 2360-2381 nt), and EV-A71-VP1-R: 5’-AGTAAGTCGCGAGAGCTGTCTTC-3′(nucleotides 3420-3443 nt); CV-A16-VP1-F: 5`-AGGTACTACACCCAGTGGTCAG-3′(nucleotides 2030-2052 nt), and CV-A16-VP1-R: 5`-GCAAGGTGCCGATTCACTACCCT-3′(nucleotides 3400-3423 nt). The partial VP1 genes of the CV-A6 and CV-A10 stains isolated were amplified by RT-PCR using primers: CV-A6-VP1-F: 5′- GGCACCCAAGCTCTCACGTG -3′(nucleotides 754-773 nt),and CV-A6-VP1-R: 5`- TTAGGGCGGTTAAGACTGGA -3`(nucleotides 2245-2264 nt); CV-A10-VP1-F: 5`-CCAGCACTGACAGCAGYNGARAYNGG-3′(nucleotides 2602–2627 nt), and CV-A10-VP1-R: 5`-TACTGGACCACCTGGNGGNAYRWACAT-3′(nucleotides 2951-2977 nt). RT-PCR reactions were performed using one-step RT-PCR kit (QIAGEN, Germany). The cycling conditions included RT step at 50 °C for 30 min, initial denaturation at 95 °C for 15 min, followed by 34 cycles of danturation at 95 °C for 30s, annealing at 52 °C for 45 s, and extension at 72 °C for 90s. A final extension at 72 °C for 10 min was also included. Negative controls replaced template by sterile water. The 5′ non-translated region of non-EV-A71/CV-A16/CV-A6/CV-A10 enterovirus (nucleotides 160-610 nt) was amplified using the RT-PCR one step kit for the Serotype determination of Enterovirus (GenTouch, Beijing, China) according to the manufacturer’s protocols, and then primers 292 and 222 for the VP1 region were used for further genotyping as described previously. The primers used for VP1 region of non-EV-A71/CV-A16/ CV-A6/CV-A10 enterovirus were as follows: 229: 5`-CICCIGGIGGIAYRWACAT-3`; 292:5`-MIGCIGYIGARACNGG-3 [25]. Amplified products were electrophoresed on 1.5% (*w*/*v*) agarose gel and visualized by UV/ethidium bromide. RT-PCR products were sent to Sangon Biotech Co., Ltd. (Shanghai, China) for DNA sequencing using an automated ABI 3730 DNA sequence (Thermo Fisher Scientific, Waltham, MA, USA).

### Phylogenetic analysis

The genetic identity of each PCR product was first determined by comparison with the reference strains in GenBank (US National Center for Biotechnology Information, NCBI), and the sequences were submitted to GenBank under accession numbers MH491075-MH491166. Multiple sequence alignment of the entire VP1 nucleotide sequences (891 bp) of the EV-A71 and CV-A16 stains and partial genes of the CV-A6 (759 bp) and CV-A10 (300 bp) were performed using Clustal W program. Phylogenetic trees were conducted in MEGA 5.0 using the neighbor-joining method in Maximum Composite Likehood model, accompanied by bootstrap analyses with 1000 replicates [26, 27]. Reference sequences representing EV-A71, CV-A16, CV-A6 and CV-A10 have downloaded from NCBI database and subjected to phylogenetic analysis together with our isolates.

### Space-time scan statistic

A retrospective space-time scan statistic was utilized to detect clustering of severe HFMD using the SaTScanTM software (http://www.satscan.org/,version 9.4, Boston, MA, USA) with a discrete Poisson model during the study. In this approach, the spatial aspect was governed by the circular scan window, which constitutes the bottom of a cylinder, whose height corresponds to the time dimension. The spatial size of scanning window was limited to 20% of the total at-risk population and the length of time limited to 3 months. The number of Monte Carlo replications was set at 999. The null hypothesis was that the disease risk inside the window was the same as outside the window, while the alternative hypothesis were that the risk was elevated inside the window compared with the outside. For each window, a log likelihood ratio (LLR) was calculated and a *p*-value estimated through Monte Carlo simulation. The window with the maximum LLR was defined as the primary cluster, other windows with statistically significant LLRs were considered as the secondary clusters.

### Statistical analysis

The severe illness rate was defined as the number of severe cases divided by the total number of the population, while the mortality rate was defined as the number of fatal cases divided by total number of the population. The case-severity rate was calculated as the number of severe cases divided by the total number of HFMD cases. The severity−fatality rates, severity-PICU admission rates, and severity-hospitalization rates were defined as the number of deaths, number of severe cases admitted to PICU, and number of hospitalized severe cases divided by the total number of severe cases, respectively. Demographic data were collected from the Scientific Data Sharing Center of Public Health (http://www.phsciencedata.cn/Share/index.jsp). We estimated 95% Confidence Intervals (CIs) with Poisson methods. The correlation coefficients was utilized to analyze the above parameters, and the EV-A71 proportion, calculated using EV-A71 positive cases was divided by all mild and severe laboratory-confirmed cases. We calculated the time distribution from onset to the initial visits, time distribution from onset to the diagnosis of severe HFMD, time distribution from onset to the hospitalization of severe HFMD by years, geographic districts and genotypes. All statistical analyses were performed using the Statistical Package for Social Sciences (SPSS; version 17.0). We used the chi-square test for analyzing categorical data. The accepted level of significance for all analyses was *P* < 0.05.

## Result

### Epidemiology and disease burden of severe HFMD cases in Jiangsu province from 2009 to 2015

#### Time distribution

**Fig. 1:**
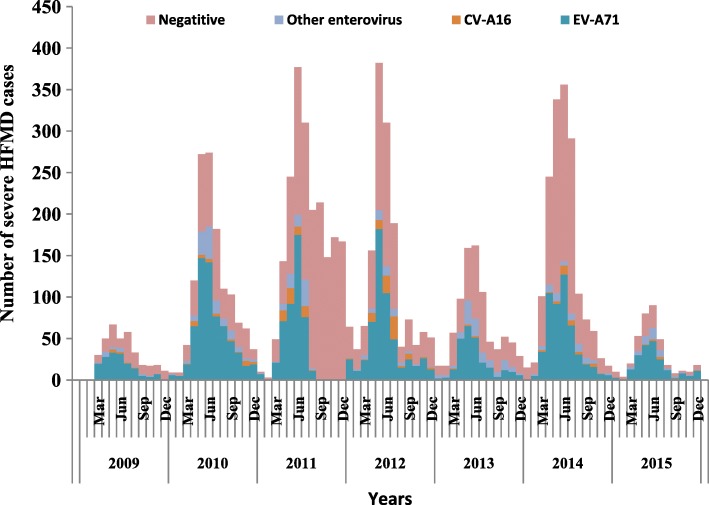
Monthly distribution of number of severe HFMD by week of illness onset during 2009–2015 in Jiangsu province

759,691 probable cases of HFMD were reported to the National Noticeable Disease Reporting System (NNDRS) in Jiangsu province during 2009–2015, of which 7994 were severe cases. Among severe cases, 7224(90.4%) cases were inpatients, 611 (7.6%) cases were PICU patients, and 68 (0.9%) were fatal. The average severe illness rate, mortality rate, severity−fatality rate, severity-PICU admission rate, and severity-hospitalization rate were 14.54, 0.12, 8506, 76,430, and 903,700 per 1 million, respectively. During the seven years period, There were two most severe outbreaks of HFMD in 2011 and 2014 with a severe illness rate of 25.97/1 million and 20.73/1 million, respectively (Fig.1, Additional file 1: Table S1). Responding to the mortality rates, severity−fatality rates, and case−fatality rates showed a gradual decline from 2010 to 2015. However, the severity-PICU admission rates were relatively lower in 2009 (33,990/ 1 million) and 2010 (44,220/1 million), compared with that of 60,210/ 1 million~ 167,300/ 1 million in the following 5 years. The severity-hospitalization rate kept steady at about 903,700/ 1 million during the whole study period of 7 years. In addition, when the monthly proportion of HFMD cases associated with EV-A71 increased, the severe illness rate, mortality rate, case-severity rate, severity−fatality rate, and case−fatality rate increased accordingly (r values ranged from 0.423 to 0.644, with *p* values < 0.001).

#### Regional distribution and spatiotemporal cluster analysis

**Fig. 2:**
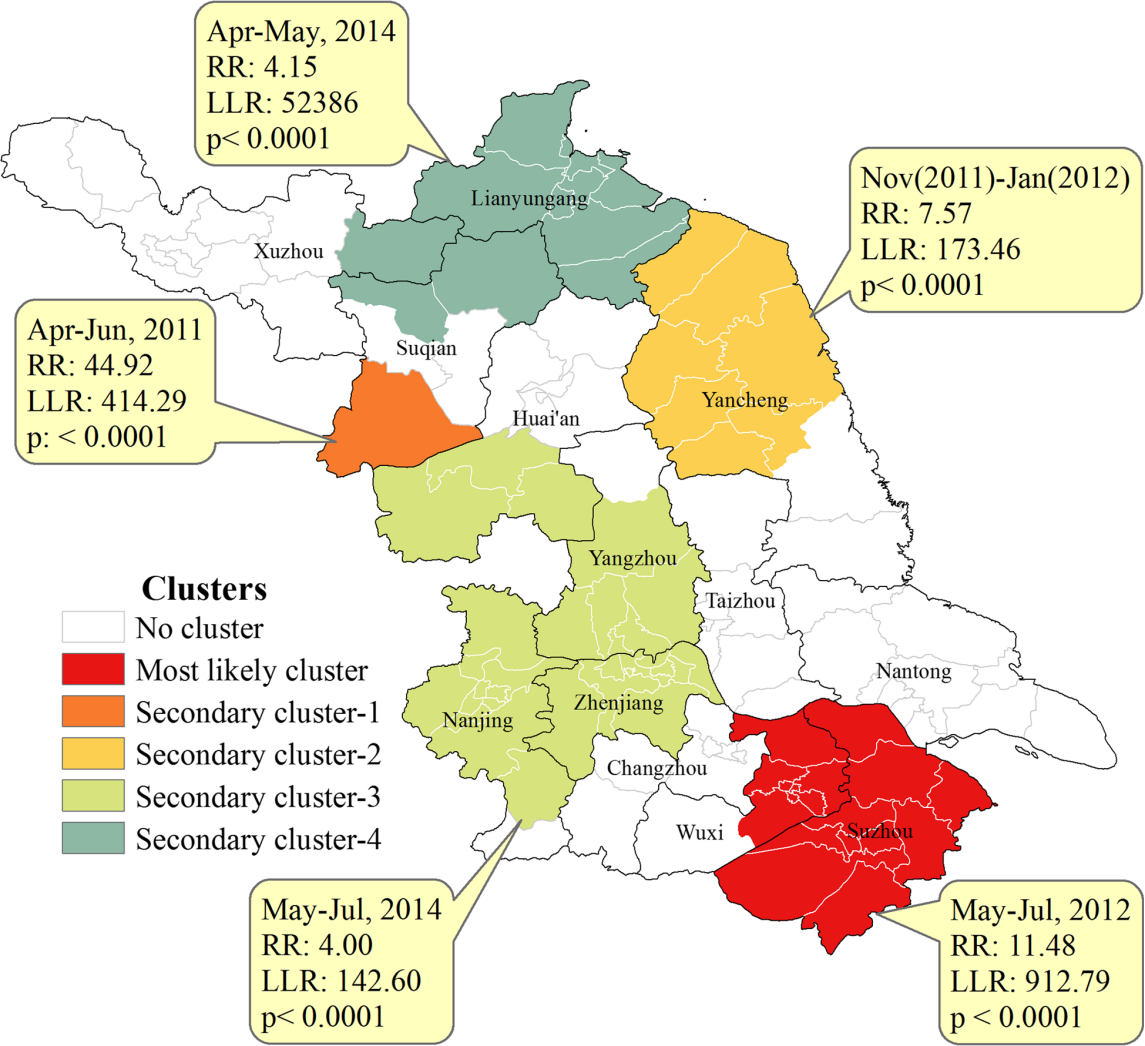
Spatiotemporal clusters of severe HFMD during 2009–2015 in Jiangsu province

Five spatiotemporal clusters of total severe cases were detected by space-time scan statistical analysis, including one primary cluster and four secondary clusters. The most likely clusters, covering 18 country townships, consisted of 611 severe cases and were located in the southern regions from May to July in 2012, with a relative risk (RR) of 11.48. The secondary clusters were mainly gathered in the northern regions. The first secondary clusters was located in northern region from April to June in 2011 with a RR of 44.92 (Fig. 2), with only one country township affected. The different variables were all statistically significant by geographic regions. Cities of southern regions including Suzhou, Wuxi, Changzhou, Nanjing, and Zhenjiang usually had the highest severe illness rates, case-severity rates and severity-hospitalization rates, but with the lowest severity−fatality rates and severity-PICU admission rates. Cities of northern regions including Yancheng, Huai^’^an, Suqian and Lianyungang demonstrated the greatest mortality rates, severity−fatality rates, case−fatality rates and severity-PICU admission rates (Table 1). There was no significant difference among the pathogens in the above three regions (χ2 = 8.740, *p* = 0.068), with EV-A71 accounting for 79.05, 83.44, 79.13% in the southern, central, and northern regions, respectively.

**Table 1:** Estimated rates in severe HFMD cases by time, regions, and population(per 1000 000 people, 95% confidence internal)^a^

Years	Severe illness rate	Mortality rates	Case-severity rates	Severity−fatality rate	Case−fatality rates	Severity-PICU admission rates	Severity-hospitalization rates
Year							
2009	4.60(4.14–5.10)	0.08(0.03–0.16)	4205(3783–4661)	17,000(6889–35,350)	71.47(28.97–148.60)	33,990(18420–57,790)	892,400(797800–995,100)
2010	16.69(15.79–17.60)	0.27(0.17–0.41)	1488(1408–1571)	16,290(10350–24,480)	242.40(154.10–364.20)	44,220(33810–56,880)	918,500(867300–972,000)
2011	25.97(24.86–27.12)	0.24(0.15–0.37)	1779(1703–1858)	9300(5765–14,250)	165.50(102.60–253.60)	60,210(50250–71,580)	882,500(842500–924,000)
2012	18.57(17.64–19.54)	0.14(0.07–2.42)	1268(1205–1334)	74,908(3943–13,030)	95.09(50.00–165.30)	79,070(65630–94,490)	909,300(861500–959,600)
2013	10.42(9.72–11.15)	0.05(0.02–0.12)	8559(7990–9158)	4848(1541–11,700)	41.05(13.19–100.10)	167,300(141100–197,000)	865,500(803700–930,700)
2014	20.73(19.75–21.75)	0.08(0.03–0.16)	9848(9380–10,330)	3645(1477–7582)	35.90(14.55–74.66)	84,450(71260–99,390)	925,300(879700–972,600)
2015	4.66(4.20–5.15)	0.01(0.00–0.06)	3903(3520–4315)	2695(134.9–13,290)	10.52(0.52–51.88)	70,080(46750–101,200)	946,100(850900–1,049,000)
Total	14.54(14.22–14.86)	0.12(0.10–0.16)	10,520(10290–10,760)(10,290–10,760)	8506(6657–10,720)	242.40(154.10–364.20)	76,430(70550–82,680)	903,700(883000–924,700)
*P value*	*p* < 0.001	*p* < 0.001	*p* < 0.001	*p* < 0.001	*p* < 0.001	*p* < 0.001	*p* < 0.001
Regions							
Southern	25.81(25.14–26.49)	0.11(0.07–0.16)	13,290(12950–13,640)	4294(2815–6292)	57.08(37.42–83.63)	4920(43640–55,280)	912,500(887700–937,800)
Central	3.41(3.08–3.75)	0.05(0.02–0.11)	2764(2502–3046)	15,110(6126–31,430)	41.77(16.93–86.89)	73,050(49850–103,500)	856,400(768900–951,200)
Northern	9.26(8.87–9.68)	0.18(0.13–0.24)	10,270(9828–10,730))	18,920(13580–25,710)	194.40(139.50–264.00)	152,900(136500–170,700)	888,400(847900–930,400)
*p value*	*p* < 0.001	*p* < 0.05	*p* < 0.001	*p* < 0.001	*p* < 0.001	*p* < 0.001	*p* < 0.001
Age groups							
< 6 months	27.52(21.83–34.25)	0.00(0.00–0.00)	14,350(11390–17,870)	0.00(0.00–0.00)	0.00(0.00–0.00)	157,900(85550–268,400)	868,400(677100–1,098,000)
6-11 months	313.9(293.5–335.4)	7.24(4.55–10.99)	11,280(10550–12,050)	23,070(14490–34,990)	260.30(163.50394.90)	144,200(120500–171,200)	899,700(838200–964,500)
12-23 months	502.1(484.3–520.5)	5.43(3.18–7.58)	14,200(13690–14,720)	10,820(7529–15,090)	153.70(106.90–214.30)	81,500(71690–92,300)	905,600(871800–940,400)
24-35 months	304.4(290.3–318.9)	1.57(0.77–2.88)	11,350(10820–11,890)	5161(2517–9470)	58.56(28.56–107.50)	59,630(48790–71,960)	897,400(853700–942,700)
36-47 months	222.6(210.5–235.3)	1.44(0.67–2.74)	8933(8446–9442)	6472(3006–12,290)	57.82(26.85–109.80)	58,250(45920–72,930)	904,500(852700–958,700)
48-59 months	114.8(106.1–124.00)	0.00(0.00–0.00)	7172(6628–7749)	0.00(0.00–0.00)	0.00(0.00–0.00)	41,270(27530–59,610)	907,900(835800–984,700)
≥60 months	0.93(0.85–1.02)	0.00(0.00–0.00)	5413(4946–5911)	0.00(0.00–0.00)	0.00(0.00–0.00)	64,050(44290–89,800)	919,400(836900–1,008,000)
*P value*	*p* < 0.001	*p* < 0.001	*p* < 0.001	*p* < 0.001	*p* < 0.001	*p* < 0.001	*P* > 0.05
Gender							
Male	18.62(18.12–19.13)	0.18(0.13–0.23)	11,140(10840–11,450)	9492(7100–12,450)	105.80(79.11–138.70)	78,460(71090–86,390)	905,500(879800–931,700)
female	10.39(10.01–10.78)	0.07(0.04–0.11)	9552(9206–9909)	6709(4159–10,280)	64.09(39.73–98.22)	72,740(63300–83,200)	900,400(866000–935,900)
*P value*	*p* < 0.001	*p* < 0.001	*P* > 0.05	*P* > 0.05	*p* < 0.001	*P* > 0.05	*P* > 0.05

^a^The specific numerator and denominator were shown in Additional file 1: Table S1

#### Population distribution

**Fig. 3:**
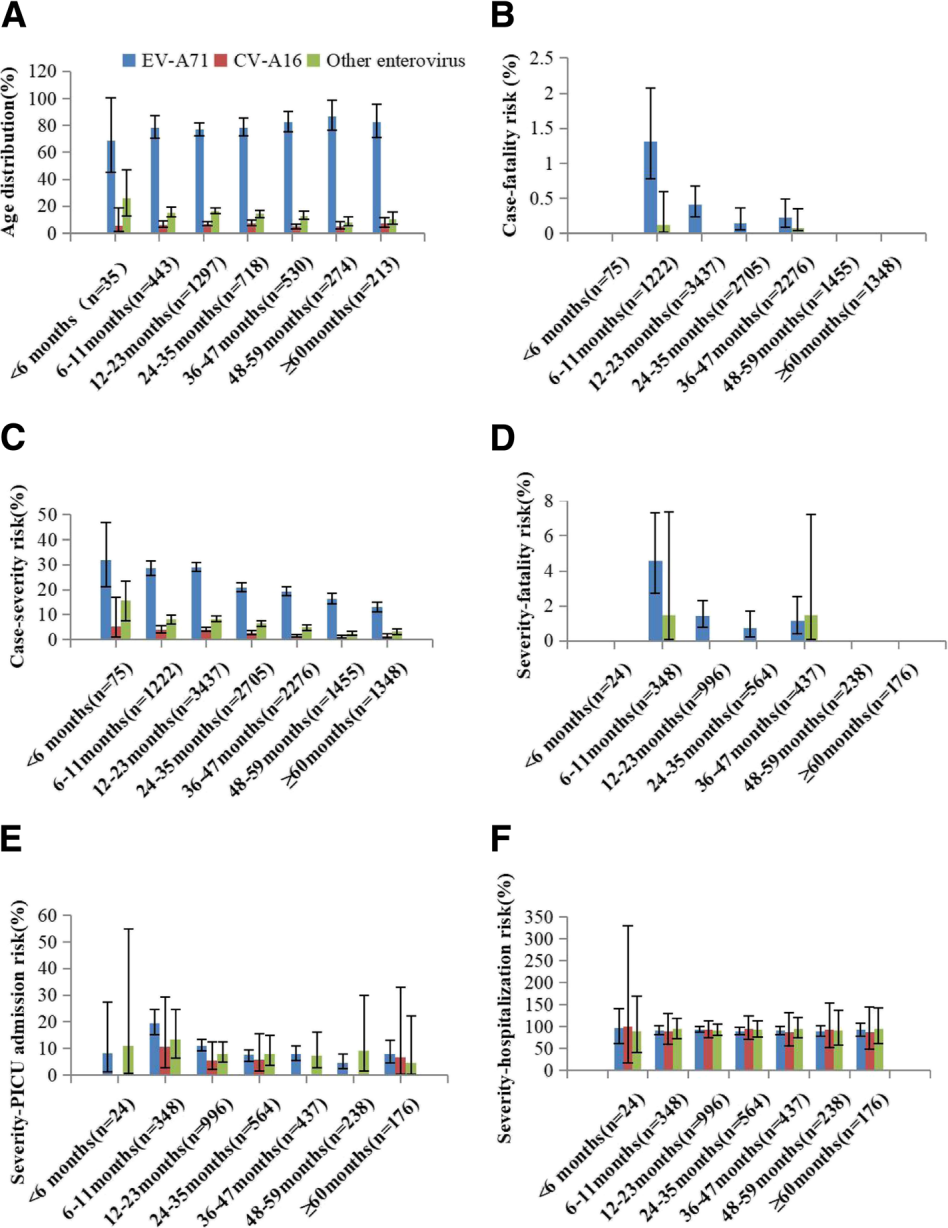
Age distribution and clinical severity of laboratory-confirmed cases of severe HFMD in Jiangsu province, 2009–2015. (**a**) Age distribution oflaboratory-confirmed severe cases. (**b**) Risk of fatality in cases by age group and serotype of virus. (**c**) Risk of severe illness in cases by age group and serotype of virus. (**d**) Risk of fatality in severe cases by age group and serotype of virus. (**e**) Risk of PICU admission in severe cases by age group and serotype of virus. (**f**) Risk of hospitalization in severe cases by age group and serotype of virus

The severity of the diseases and deaths were inversely related to age. The median age of all severe cases was 24 months (IQR:16–39 months). Most severe cases occurred in children younger than 5 years old. The highest severe illness rate of 502.1/1 million appeared in the 12 to 23 months age group, while the rate for the 24–35 months age group, for 36–47 months age group, for 48–59 months age group, and over 60 months age group were 304.4/1 million, 222.6/1 million 114.8/1 million, and 0.93/1 million, respectively. Similarly, the 6–11 months age group was associated with the greatest mortality rate, severity-fatality rate and case-fatality rate, while the risk of death decreased as the age increased in the older age group. However, no fatal case of HFMD was found in infants younger than 6 months. The severity-PICU admission rate also decreased as age increasing, demonstrating a peak rate in infants aged younger than 11 months age group. The severity-hospitalization rates remained relatively steady among different age groups, and no significant difference was observed (Table 1). Patients infected with EV-A71 were at higher risk for severe even death compared with those infected with CV-A16 and other EVs (Fig. 3b–e). The risk of hospitalization in severe cases was basically consistent among different age groups and serotypes of virus (Fig. 3f). During the 7-year study period, the severe illness rate, mortality, and case-fatality rate were all significantly higher in males than in females. However, no significant difference was found among the severity-fatality rates, case-fatality rates, severity-PICU admission rates, and severity-hospitalization rates (Table 1), and also no significant difference was found between gender and genotypes (χ2 = 0.571, *p* = 0.752), with EV-A71 accounting for 79.59% in male and 78.59% in female.

#### The time interval of onset-to-initial visits, onset-to-diagnosis, and onset-to-hospitalization

The median time interval from onset to the first clinic visit was 0.0 days (IQR 0.0–1.0 days), 3.0 days (IQR 2.0–4.0 days) from onset to diagnosis of severe HFMD, and 2.0 days (IQR1.0–3.0 days) from onset to hospitalization. The median time from onset to death of severe HFMD cases was 5.0 days (IQR3.0–7.0 days), 1.0 days (IQR0.0–3.0 days) for the diagnosis of severe HFMD to death. The time interval from onset to the initial visits was longer in 2009 and 2010 compared with the following 5 years, and the time interval from onset to the hospitalization of severe HFMD was longer in northern region than that in southern region and central region. Significant differences were found in the time intervals from onset to initial visits,onset to diagnosis, and onset to hospitalization by different years, regions, and viral genotypes(*p* < 0.05)(Table 2).

**Table 2:** The time interval of severe HFMD by different years and regions. Days(IQR)

Variables	Time from onset to the first clinic visits	Time from onset to diagnosis of severe HFMD	Time from onset to the hospitalization of severe HFMD
Year			
2009	2.0(0.0–1.0)	2.0(1.0–4.0)	3.0(2.0–3.0)
2010	1.0(0.0–1.0)	3.0(2.0–4.0)	2.0(2.0–3.0)
2011	0.0(0.0–1.0)	3.0(2.0–4.0)	2.0(1.0–3.0)
2012	0.0(0.0–1.0)	3.0(2.0–4.0)	2.0(1.0–4.0)
2013	0.0(0.0–1.0)	3.0(2.0–4.0)	2.0(1.0–3.0)
2014	0.0(0.0–1.0)	3.0(2.0–4.0)	2.0(1.0–3.0)
2015	0.0(0.0–1.0)	3.0(2.0–4.0)	2.0(1.0–3.0)
District			
South-region	0.0(0.00–1.00)	3.0(1.0–4.0)	2.0(1.0–3.0)
Central-region	1.0(0.0–1.00)	3.0(2.00–4.0)	2.00(1.0–4.00)
North-region	0.0(0.0–1.0)	3.0(2.0–5.0)	3.00(1.0–4.0)
Pathogen			
EV71	0.0(0.0–1.0)	3.0(2.0–4.0)	2.0(1.0–3.0)
CVA16	0.0(0.0–1.0)	2.0(1.5–4.0)	2.0(1.0–3.0)
Other enteroviruses	0.0(0.0–1.0)	3.0(2.0–4.0)	2.0(1.0–3.0)

#### Central nervous system complications by different serotype of virus

Among the 3510 laboratory-confirmed severe cases, 3469 patients survived after treatment. Of the survived patients, 1314(37.44%) were diagnosed with aseptic meningitis, 2092(59.60%) with encephalitis, 40 (1.14%) with encephalomyelitis, 11(0.31%) with pulmonary oedema/ haemorrhage, 3 (0.09%) with acute flaccid paralysis, 3 (0.09%) with brainstem encephalitis, and 6(0.18%) with cardio-respiratory failure. Patients infected with EV-A71 were at higher proportion in different CNS involvements and even deaths related to severe cases (Table 3).

**Table 3:** Detailed numbers of cases showing each type of neurological complication associated with different serotype of virus of severe HFMD (*N* = 3510) ^a^

CNS^b^ complications	EV-A71	CV-A16	Other EVs	Total	x^2^	p
aseptic meningitis(Survivors)	1088(31.40)	95(2.71)	131(3.73)	1314(37.44)	54.47	0.000
encephalitis(Survivors)	1604(45.70)	125(3.56)	363(10.34)	2092(59.60)		
Encephalomyelitis(Survivors)	33(0.94)	3(0.09)	4(0.11)	40(1.14)		
Pulmonary oedema/ haemorrhage	10(0.28)	0(0.00)	1(0.03)	11(0.31)		
Acute flaccid paralysis(Survivors)	2(0.06)	0(0.00)	1(0.03)	3(0.09)		
Brainstem encephalitis(Survivors)	3(0.09)	1(0.03)	0(0.00)	3(0.09)		
Cardiorespiratory failure(Survivors)	4(0.11)	2(0.06)	0(0.00)	6(0.17)		
Death	39(1.11)	0(0.00)	2(0.06)	41(1.17)		
Total	2783(79.29)	225(6.41)	502(14.30)	3510(100.00)		

^a^Data outside the parentheses are the number of severe HFMD cases, data in parentheses are % (n/N)

^b^CNS: Central nervous system

#### Pathogen spectrum of severe HFMD cases

**Fig. 4:**
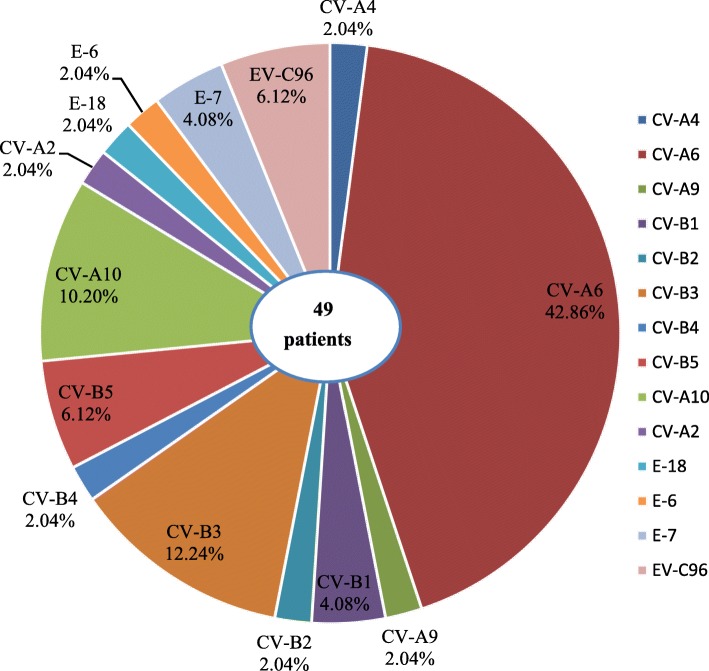
The genotypes of non-EV-A71/CV-A16 enteroviruses identified in severe HFMD patients

Among 3510laboratory confirmed severe HFMD cases, with 2783(79.29%), 225(6.41%), and 502(14.30%) associated with EV-A71, CV-A16, and other EVs, respectively. Of the 3221 hospitalized severe HFMD cases confirmed by laboratory testing, EV-A71, CV-A16 and other EVs accounted for 79.14, 6.49, and 14.47%, respectively. Of the 336 laboratory-confirmed severe HFMD cases admitted to PICU, EV-A71, CV-A16, and other EVs accounted for 83.63, 3.57, and 12.80%, respectively. Of the 41 fatal cases confirmed by laboratory testing, EV-A71 and other EVs accounted for 95.12 and 4.88%, respectively. Furthermore, a total of 14 genotypes were identified by sequencing in 49 patients who were infected with other EVs (9.76%, 49/502) from 2012 to 2015. The most prevalent genotype was CV-A6(42.9%, 21/49), followed by CV-B3(12.2%,6/49), CV-A10(10.2%, 5/49), EV-C96(6.1%,3/49), E-7(4.1%,2/49), etc. (Fig. 4). The results indicated that various EVs circulated and caused severe HFMD cases in Jiangsu province.

#### Phylogenetic analysis of the VP1 genes of EV-A71, CV-A16, CV-A6, and CV-A10

To characterize the EV-A71, CV-A16, CV-A6 and CV-A10 strains circulating in Jiangsu province and investigate their genetic origin, we analyzed the VP1 genes of viruses isolated from clinically diagnosed with severe HFMD patients’ specimens. A total of 47 EV-A71 strains (isolated from 43 severe-survivors and 4 deaths), 19 CV-A16 strains, 21 CV-A6 strains, and 5 CV-A10 strains were isolated. The nucleotide sequences of EV-A71 isolates in our study were closely related to each other, sharing 91.7 to 100.0% nucleotide identity and corresponding to 98.5 to 100.0% amino acid identity. The 145E of VP1 was a predominant form in both severe and fatal cases. A similar analysis from CV-A16 isolates showed 87.9 to 100.0% nucleotide identity, corresponding to a 99.0 to 100.0% amino acid identity. The nucleotide and amino acid identities in severe CV-A6 strains were 91.1–100.0% and 97.1–100.0%, while the nucleotide and amino acid identities in CV-A10 were 92.0–100.0% and 99.0–100.0%, respectively.

**Fig. 5:**
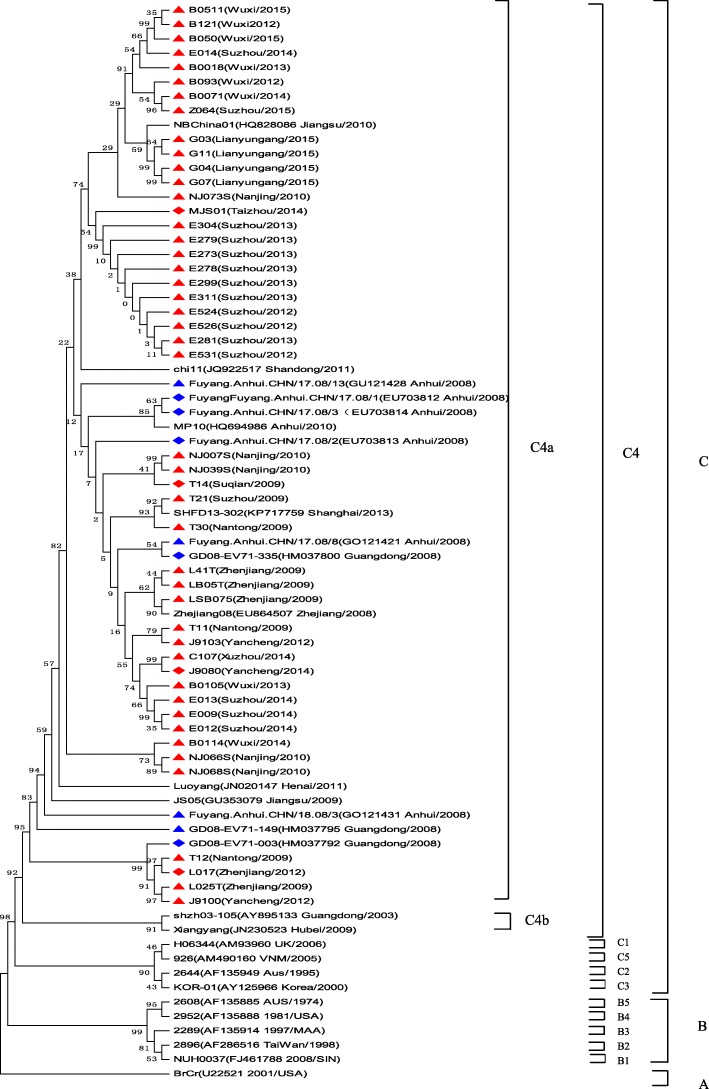
Phylogenetic tree based on the complete VP1 sequences of Jiangsu EV-A71 isolates and reference strains of severe and fatal HFMD cases. (Strains indicated by red solid triangle indicated the severe cases, Red solid diamond indicated the fatal cases in this study, EV-A71 strains isolated from severe cases and fatal cases in other cities were indicated by a blue solid triangle and a blue solid diamond, respectively)

**Fig. 6:**
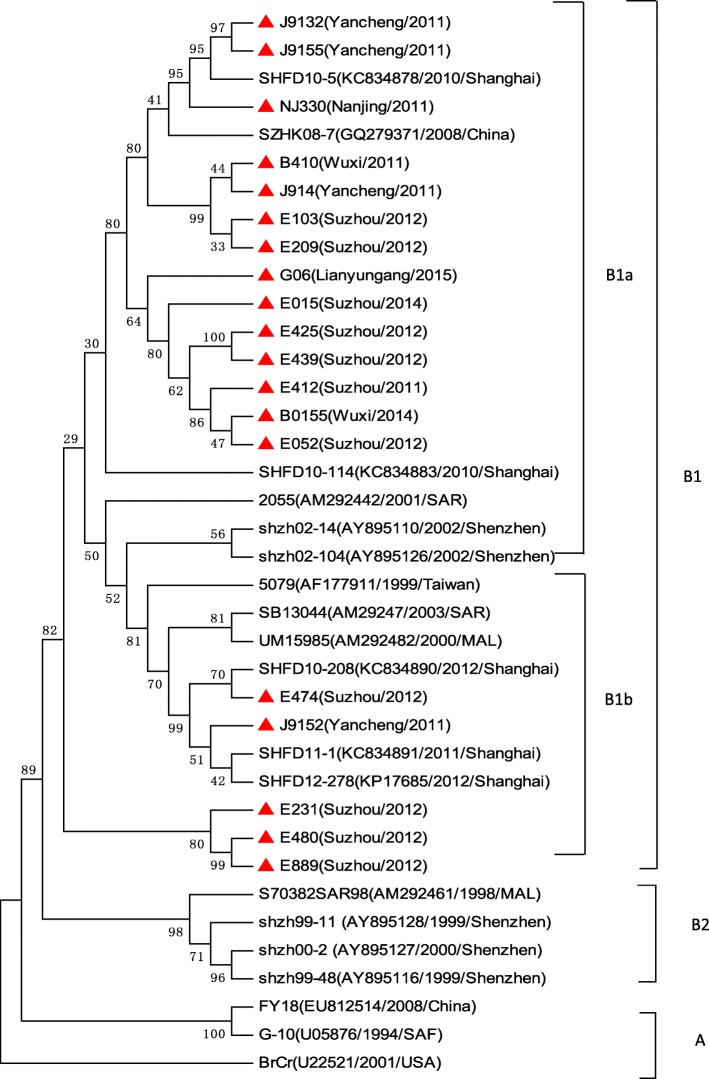
Phylogenetic tree based on the complete VP1 sequences of Jiangsu CV-A16 isolates and reference strains of severe HFMD cases. (Strains indicated by red solid triangle are CVA-16 strains that were isolated from severe cases)

**Fig. 7:**
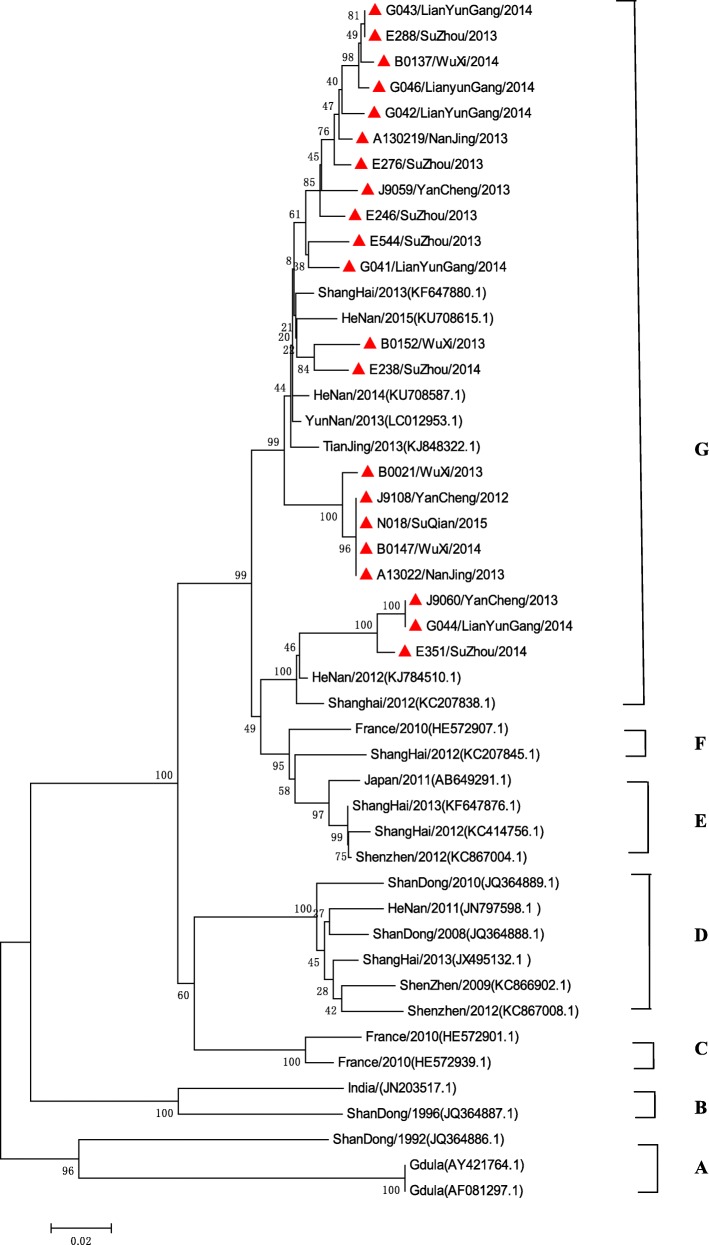
Phylogenetic tree based on the partial VP1 sequences of Jiangsu CV-A6 isolates and reference strains of severe HFMD cases

**Fig. 8:**
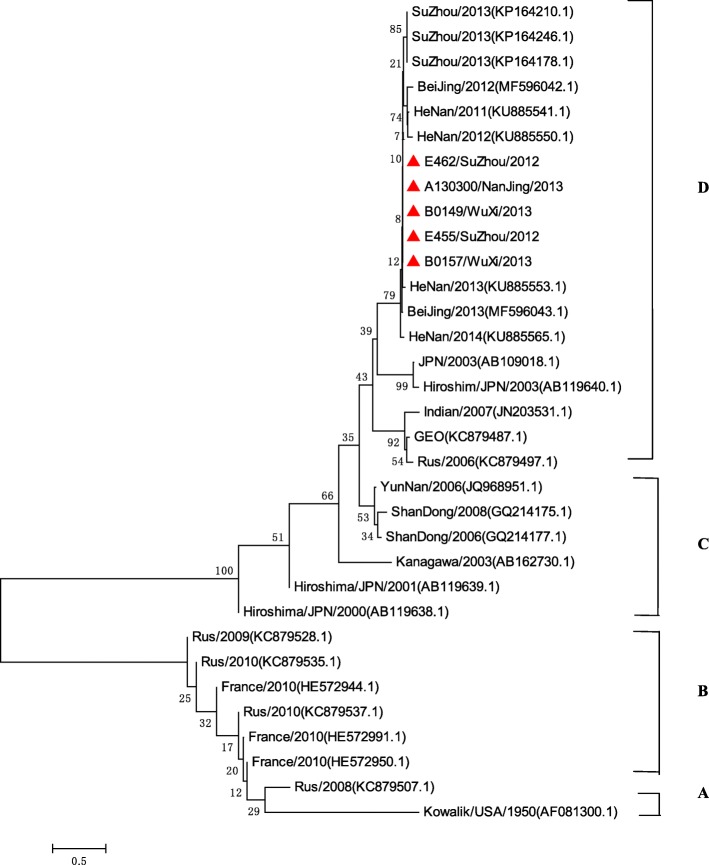
Phylogenetic tree based on the partial VP1 sequences of Jiangsu CV-A10 isolates and reference strains of severe HFMD cases

A total of 75 EV-A71 strains, 38 CV-A16 strains, 47 CV-A6 strains, and 33 CV-A10 strains were used for phylogenetic analyses. All 47 EV-A71 strains from Jiangsu Province clustered exclusively to genotype C, subtype 4a (C4a) (Fig. 5). The 19 nucleotide sequences of CV-A16 were closest in the B1 gene subtype, belonged to clusters B1a and B1b (Fig. 6). 21 CV-A6 isolates in this study from 2012 to 2015 were all located in cluster G (Fig. 7), 5 CV-A1 isolates were all located in cluster D (Fig. 8).

## Discussion

In this study, we found the severe illness rates peaked periodically with an increase in 2011 and 2014, suggesting that severe HFMD epidemics presented a periodic circulation every 2–3 years, which was in consistent with the previous reports in Malaysia and Singapore [28, 29]. The mortality and severity-fatality rates presented with decreasing tendency during the study period. However, the severity-PICU admission rates increased from 2009 to 2013 and peaked in 2013, and then maintained at a high level in 2014 and 2015. The severity-hospitalization rates remained steady. This phenomenon may due to the following reasons: Firstly, EV-A71 infection is more commonly associated with severe HFMD cases and fatalities, but the annual incidence of EV-A71 seroconversion is no more than 10% with an accumulation of susceptible young children, which allowing an accumulation of the susceptible individuals [30]. Another possible reason is that the designation of specific hospitals for treatment of severe cases has implemented since 2010. The third reason is attributed to the medical service response, such as clinical monitoring for the early detection and intervention of severe cases. Besides, guidelines have been developed for the treatment of severe HFMD cases, and PICU guideline has also been established and strengthened. Furthermore, the mortality of HFMD in our study was higher than that reported in mainland China from 2008 to 2014 [30], which may indicate a more severe prevalence of HFMD in Jiangsu Province. However, according to Xueyong Huang’s report, the number of severe and death cases (22,309 severe cases and 141 death) observed in Henan province from 2008 to 2013 were even higher [21]. Therefore, these data showed that the prevalence of HFMD in different region of China varied, which may be associated with the differences in circulation of virus, population density, level of diagnosis and treatment in different regions.

This study found that the severe illness rates and mortality were much higher in the young children, especially in the 6–23 months age group, which were similar to the report of other studies in mainland China and Taiwan [18, 20]. Thus, more concerns were needed to focus on this age group. Relatively lower severe illness rates and death risk were found in infants younger than 6 months, which were most likely due to the maternal antibodies to be able to protect infants against severe outcomes of infection [18, 31, 32]. Nevertheless, this age group was at higher case-severity rates, PICU admission rates and hospitalization rates. The youngest infants allowed to get EV-A71 vaccine were those at age 6 months [33, 34], which leaves young infants vulnerable. Future studies may focus on the vaccination at birth and at 2, 3, or 4 months to optimize protection. Furthermore, clinical trials showed that EV-A71 vaccines manufactured in China were highly efficacious against EV-A71-associated hospitalization and on HFMD with neurologic complications in infants and young children [34]. More surveillance efforts on the characteristic of severe HFMD should be made after inactivated EV-A71 whole virus vaccine vaccination widespread in the future. In relation to gender, males were at higher the severe illness rates and mortality, which has also been observed in other reports [20, 29]. However, the chance of progressing to hospitalization, PICU admission, and even death was identical, detailed study of host genetic level, host immune status and/or behavior patterns would be needed to clarify.

The time interval from onset to the first clinic visit was longer in 2009 and 2010 than that in other years, and the mortality and severity-fatality rates were also relatively higher in the corresponding years. Furthermore, we found that the time interval from onset to the hospitalization was the longest along with the elevated death risk in northern region of Jiangsu, which indicated that early recognition and timely intervention was the keys to reduce acute morbidity and mortality associated with severe presentation. In addition, southern regions usually had the highest severe illness rates, case-severity rates and severity-hospitalization rates, whereas northern regions had the highest mortality rates, severity-fatality rates, case-fatality rates and the severity-PICU admission rates, which suggested that it worthy of conducting a special research to understand the underlying causes to employ more targeted control strategies for this disease. Future mitigation policies should take into account the region heterogeneities of disease burden idendified.

Our study showed that it was more likely for EV-A71 infection than other enterovirus types to cause serious CNS and EV-A71 usually led to encephalitis, pulmonary oedema/ haemorrhage, circulation failure and death, which was in line with other published studies on EV-A71 infection [17, 20]. The involvement of different parts of the CNS may lead to different outcomes including neurologic and phychiatric effects [35]. Our research results could probably help us master the severe clinical symptom spectrum and the disease severity burden.

Previous studies showed that all known EV-A71 strains could be classified into three genotypes-A, B and C [36]. Genotypes B and C are further divided into subgenotypes B1 to B5 and C1 to C5, respectively [5, 36], the subgenotype C4 could be further divided into C4a and C4b clusters [37]. According to the phylogenetic analyses, Jiangsu isolates belonged to C4a cluster, which was most closely related to the strains isolated in China [30, 38], indicating far less variety in mainland China. By contrast, changes in the predominant subgenogroup are also observed at each epidemic wave in Taiwan and Singapore [5], along with the different epidemic intensity of HFMD [29, 39]. It was interesting to note that Chang et al. reported the VP1-145E variants were mainly responsible for the development of neuropathogenesis in EV-A71-infected individuals, which was also showed in our study. Most study demonstrated the minority of CV-A16 associated with severe HFMD, our study supported this observation, CV-A16 has been grouped into genogroups A, B1a, B1b and B2 [40]. Our study revealed co-circulation of B1a and B1b, the result was different from the previous study, which was only identified as B1b subgenogroup in 2012–2013 in Shanghai city, with no significant variation from the strains isolated in other areas of China [38, 41].

Diversities of serotypes of non-EV-A71 /CV-A16 enteroviruses associated with severe HFMD were rarely reported in other previous studies. Our findings suggested that besides of EV-A71 and CV-A16, a total of 14 enterovirus genotypes were associated with severe HFMD cases, and the higher detection rate of CV-A6 was found in our study. In recent years, CV-A6 has caused some outbreaks in Finland [42], Taiwan [43], the United States [44], Japan [15], and China [45], while with the CV-A6 becoming another major pathogen of HFMD, patients infected with CV-A6 had central nervous system (CNS) involvement were also reported [13, 15, 45–47]. Previous study reported CV-A6 infections attacked a broader spectrum of skin sites, caused more profound tissue damages [43], and the higher hospitalization rate [48]. In our study, isolated strains from severe cases of CV-A6 were all located on the same cluster of the phylogenetic tree, indicating that they shared the single ancestor and were undistinguishable in Jiangsu province, perhaps due to the the same genotype prevalent during the study period. A descriptive study revealed that CV-A10 was independently associated with higher risk of severe HFMD [19]. Our study found all the severe CV-A10 isolates belonged to genogroup D, which mainly included isolates from mainland China [49]. Yang F found that CV-A10 and CV-A4 in one severe case each [47]. CV-A2 and CV-A9 have also been associated with the outbreaks of HFMD, and CV-A9 was one of the EVs that may be associated with aseptic meningitis. CV-B1 was the predominant enterovirus isolated in the USA among young infants with severe disease in 2007 [50] and continued to be the most common serotype detected in 2008. CV-B2, CV-B3 and CV-B5, associated with neurological HFMD have also been reported [10, 51, 52]. CV-B4 was one of the highest rates of fatal outcome (9.8‰) [53, 54], and also been isolated from a patient with a fatal HFMD disease in China [55]. Most previous reports of E-6 infections indicated that the virus mainly causes the aseptic meningitis outbreak [56–59]. E-6 associated with HFMD has also been reported in China, but without detail information about the clinical manifiestion [14]. E-7 infection association with CNS manifestations of HFMD patients had been reported [60]. A few reports showed E-18 associated with aseptic meningitis outbreak [61, 62], while some reported E-18 was associated with some mild HFMD cases in Jiangsu province [10]. In our study, E-18 was also found to be associated with severe HFMD patients. EV-C96 has been isolated from patients with AFP and healthy individuals in Yunnan, Guangdong, and Shandong province, [63–65]. In this study, we firstly found EV-C96 infection was associated with severe HFMD. Careful surveillance of clinical manifestion, infectious agent activities and pathogenic features is crucial in monitoring non-EV-A71/ CV-A16 virus infection associated with severe HFMD of neurological complications. While the HFMD vaccine was developed for EV-A71, these pathogenic compositions of severe HFMD might affect future vaccine development and clinical management.

This study has several limitations. Firstly, accessing to and provision of health care and technical capacity varied between and sometimes within different regions, with no formal quality assurance or systematic audit for disease surveillance. Secondly, the number of isolated virus sequenced for EV-A71, CV-A16, non-EV-A71/ CV-A16 EVs of severe HFMD was small, therefore, virological surveillance data should be interpreted with caution. If more EVs isolated were routinely sequenced, we might have a better understanding of the EVs of the virus in severe HFMD. Thirdly, some severe neurological illnesses caused by EV-A71 might have been missed by using a strict HFMD case definition. Fourth, our study was based on a descriptive analysis of surveillance data. Thus, we can not identify the differences on epidemical or etiological characteristics between the severe and mild HFMD cases, which may need further studies..

## Conclusions

In conclusion, this is the first study demonstrating the comprehensive account of the burden, epidemiology,and CNS complications in severe HFMD in Jiangsu Province, China. The occurrence of severe HFMD in Jiangsu province was closely related to time, age, and region distribution. Furthermore, EV-A71 infection conferred the most serious risks of clinical severity and fatality. Additionally, we showed that the dominant subtypes of EV-A71 was C4a, the dominant subtypes of CV-A16 were B1a and B1b, the dominant subtype of CV-A6 was G, and the dominant subtype of CV-A10 was D. Finally, 12 non-EV-A71 /CV-A16/CV-A6/CV-A10 EVs associated with severe HFMD were detected in Jiangsu province. Further a more rigorous study of case control or cohort design between mild and severe HFMD disease should be badly needed to optimize the choice of interventions.

## Additional file

Additional file 1: **Table S1.** Specific parameters for calculating the rates in severe HFMD cases by time, regions, and population. (DOC 66 kb)

## Abbreviations

AFP: Acute flaccid paralysis
CNS: Central nervous system
CPE: Cytopathic effect
CV-A16: Coxsackievirus A16
E: Echovirus
EV-A71: Enterovirus A71
EVs: Enteroviruses
FBS: Fetal bovine serum
HFMD: Hand, foot, and mouth disease
LLR: Log likelihood ratio
MEM: Minimum essential medium
PICU: Pediatric intensive care unit
RD: Rhabdomyosarcoma
