# Supplementary material for: Surveillance for severe hand, foot, and mouth disease from 2009 to 2015 in Jiangsu province: epidemiology, etiology, and disease burden
Source: BMC Infect Dis. 2019 Jan 22;19:79. doi: 10.1186/s12879-018-3659-7 (PMC6341624; doi:10.1186/s12879-018-3659-7)
Supplement: Supplementary file 1 — Table S1. Specific parameters for calculating the rates in severe HFMD cases by time, regions, and population. (DOC 66 kb) [file 12879_2018_3659_MOESM1_ESM.doc]

| **Years** | **Severe illness rates** | **Mortality rates** | **Case-severity rates** |  | **Severity−fatality rate** |  | **Case−fatality rates** |  | **Severity-PICU admission rates** |  | **Severity-hospitalization rates** |
| --- | --- | --- | --- | --- | --- | --- | --- | --- | --- | --- | --- |
| **Year** |  |  |  |  |  |  |  |  |  |  |  |
| 2009 | 353/76772916 | 6/76772916 | 353/83955 |  | 6/353 |  | 6/83955 |  | 12/353 |  | 315/353 |
| 2010 | 1289/77250011 | 21/77250011 | 1289/86634 |  | 21/1289 |  | 21/86634 |  | 57/1289 |  | 1184/1289 |
| 2011 | 2043/78659857 | 19/78659857 | 2043/114825 |  | 19/2043 |  | 19/114825 |  | 123/2043 |  | 1803/2043 |
| 2012 | 1467/78987984 | 11/78987984 | 1467/115680 |  | 11/1467 |  | 11/115680 |  | 116/1467 |  | 1334/1467 |
| 2013 | 825/79199822 | 4/79199822 | 825/96391 |  | 4/825 |  | 4/96391 |  | 138/825 |  | 714/825 |
| 2014 | 1646/79394900 | 6/79394900 | 1646/167143 |  | 6/1646 |  | 6/167143 |  | 139/1646 |  | 1523/1646 |
| 2015 | 371/79600592 | 1/79600592 | 371/95063 |  | 1/371 |  | 1/95063 |  | 26/371 |  | 351/371 |
| Total | 7994/549866082 | 68/549866082 | 7994/759691 |  | 68/7994 |  | 68/759691 |  | 611/7994 |  | 7224/7994 |
| **Regions^*^** |  |  |  |  |  |  |  |  |  |  |  |
| Southern | 5589/216542953 | 24/216542953 | 5589/420494 |  | 24/5589 |  | 24/420494 |  | 275/5589 |  | 5100/5589 |
| Central | 397/116570261 | 6/116570261 | 397/143631 |  | 6/397 |  | 6/143631 |  | 29/397 |  | 340/397 |
| Northern | 2008/216752868 | 38/216752868 | 2008/195519 |  | 38/2008 |  | 38/195519 |  | 307/2008 |  | 1784/2008 |
| **Age groups** |  |  |  |  |  |  |  |  |  |  |  |
| <6 months | 76/2761729 | 0/2761729 | 76/5295 |  | 0/76 |  | 0/5295 |  | 12/76 |  | 66/76 |
| 6-11months | 867/2761729 | 20/2761729 | 867/76835 |  | 20/867 |  | 20/76835 |  | 125/867 |  | 780/867 |
| 12-23months | 2957/5888856 | 32/5888856 | 2957/208252 |  | 32/2957 |  | 32/208252 |  | 241/2957 |  | 2678/2957 |
| 24-35months | 1744/5730183 | 9/5730183 | 1744/153696 |  | 9/1744 |  | 9/153696 |  | 104/1744 |  | 1565/1744 |
| 36-47months | 1236/5551866 | 8/5551866 | 1236/138358 |  | 8/1236 |  | 8/138358 |  | 72/1236 |  | 1118/1236 |
| 48-59months | 630/5488992 | 0/5488992 | 630/87836 |  | 0/630 |  | 0/87836 |  | 26/630 |  | 572/630 |
| ≥60months | 484/521682727 | 0/521682727 | 484/89419 |  | 0/484 |  | 0/89419 |  | 31/484 |  | 445/484 |
| **Gender** |  |  |  |  |  |  |  |  |  |  |  |
| Male | 5162/277244337 | 49/277244337 | 5162/463218 |  | 49/5162 |  | 49/5162 |  | 405/5162 |  | 4674/5162 |
| female | 2832/272621745 | 19/272621745 | 2832/296473 |  | 19/2832 |  | 19/2832 |  | 206/2832 |  | 2550/2832 |

**Supplementary Table 1: Specific parameters for calculating the rates in severe HFMD cases by time, regions, and population^★^.**

* 47 HFMD cases were missing by different regions due to unknown addresss.
